# Clinical impact of implementing humidified high-flow nasal cannula on interhospital transport among children admitted to a PICU with respiratory distress: a cohort study

**DOI:** 10.1186/s13054-021-03620-7

**Published:** 2021-06-06

**Authors:** Shinya Miura, Kazue Yamaoka, Satoshi Miyata, Warwick Butt, Sile Smith

**Affiliations:** 1 Paediatric Intensive Care Unit, The Royal Children’s Hospital Melbourne, 50 Flemington Road, Parkville, VIC 3052 Australia; 2 Teikyo University Graduate School of Public Health, Tokyo, Japan; 3 Murdoch Children’s Research Institute, Parkville, Australia; 4 Department of Paediatrics, University of Melbourne, Parkville, Australia

**Keywords:** High-flow nasal cannula, Transport, Length of stay, Respiratory support, Non-invasive ventilation

## Abstract

**Background:**

There is a limited evidence for humidified high-flow nasal cannula (HHFNC) use on inter-hospital transport. Despite this, its use during transport is increasing in children with respiratory distress worldwide. In 2015 HHFNC was implemented on a specialized pediatric retrieval team serving for Victoria. The aim of this study is to investigate the effect of the HHFNC implementation on the retrieval team on the paediatric intensive care unit (PICU) length of stay and respiratory support use.

**Methods:**

We performed a cohort study using a comparative interrupted time-series approach controlling for patient and temporal covariates, and population-adjusted analysis. We studied 3022 children admitted to a PICU in Victoria with respiratory distress January 2010–December 2019. Patients were divided in pre-intervention era (2010–2014) and post-intervention era (2015–2019).

**Results:**

1006 children following interhospital transport and 2016 non-transport children were included. Median (IQR) age was 1.4 (0.7–4.5) years. Pneumonia (39.1%) and bronchiolitis (34.3%) were common. On retrieval, HHFNC was used in 5.0% (21/420) and 45.9% (269/586) in pre- and post-intervention era. In an unadjusted model, median (IQR) PICU length of stay was 2.2 (1.1–4.2) and 1.7 (0.9–3.2) days in the pre- and post-intervention era in transported children while the figures were 2.4 (1.3–4.9) and 2.1 (1.2–4.5) days in non-transport children. In the multivariable regression model, the intervention was associated with the reduced PICU length of stay (ratio 0.64, 95% confidential interval 0.49–0.83, *p* = 0.001) with the predicted reduction of PICU length of stay being − 10.6 h (95% confidential interval − 16.9 to − 4.3 h), and decreased respiratory support use (− 25.1 h, 95% confidential interval − 47.9 to − 2.3 h, *p* = 0.03). Sensitivity analyses including a model excluding less severe children showed similar results. In population-adjusted analyses, respiratory support use decreased from 4837 to 3477 person-hour per year in transported children over the study era, while the reduction was 594 (from 9553 to 8959) person-hour per year in non-transport children. With regard to the safety, there were no escalations of respiratory support mode during interhospital transport.

**Conclusions:**

The implementation of HHFNC on interhospital transport was associated with the reduced PICU length of stay and respiratory support use among PICU admissions with respiratory distress.

**Supplementary Information:**

The online version contains supplementary material available at 10.1186/s13054-021-03620-7.

## Introduction

Children experiencing unplanned paediatric intensive care unit (PICU) admission with respiratory distress after interhospital transport is an important cohort worldwide in terms of the admission number and patient outcome [1, 2]. Unplanned PICU admission following interhospital transport accounted for 30–35% of entire PICU admission, and is associated with increased rate of invasive ventilation use and prolonged PICU stay [3–5]. Respiratory distress is the most common etiology (40–60%) in this cohort [4–7], hence suggesting that 10–15% of entire PICU admissions are unplanned and transported children with respiratory distress.

Since early-2010, humidified high-flow nasal cannula (HHFNC) has been increasingly used in PICUs and emergency departments for children with respiratory distress worldwide by the virtue of high patient comfort, safety, simplicity in its application [8]. The shortened duration of invasive ventilation was demonstrated in a study regarding the implementation of HHFNC on a PICU [9]. Recently HHFNC has been used in more various settings such as ward and transport [10–13]. Previous studies have reported the safety of HHFNC use during interhospital transport [14], and a reduced requirement of invasive ventilation during transport [7]. However, the data regarding the improved outcome of transported children by the implementation of HHFNC such as the length of PICU stay and hospital stay are lacking despite its increasing use on transport, This knowledge gap is especially important when considering the social situation that children and family are restricted in the tertiary hospital away from home, and the substantial number of critically-ill children with respiratory distress worldwide. Hence, in this study we hypothesized that the implementation of HHFNC on a transport team could lead to improved patient outcomes.

Therefore, we performed this study with the primary aim of investigating the effect of implementing HHFNC on a specialized pediatric retrieval service on the PICU length of stay. Secondary aims were to explore its effect on invasive and non-invasive ventilation use, the safety of HHFNC use during retrieval, and hospital length of stay.

## Methods

### Study design

This is a retrospective cohort study designed to estimate the clinical impact of the implementation of HHFNC on interhospital transport in late-2014–2015. This study was performed in the Paediatric Infant Perinatal Emergency Retrieval (PIPER), and PICU in the Royal Children’s Hospital (RCH), Melbourne. The study was approved by the Royal Children’s Hospital Melbourne Human Research Ethics Committee (HREC no; QA/64729/RCHM-2020). The analytical framework was illustrated to visualize the study design (Additional file 1).

### Study setting

PIPER is a specialized pediatric retrieval team who is responsible for all interhospital transport of critically-ill children < 18 years old in Victoria. In Victoria, all critically-ill children were transferred to one of two tertiary PICUs at RCH and Monash Medical Center (MMC), Clayton. The destination has been decided based on the preset catchment. The number of transported children to each hospital with respiratory distress has been comparable (Additional file 2: Fig. S7).

### Implementation of HHFNC on transport

HHFNC was implemented in the paediatric retrieval team in late-2014–2015. The protocol for HHFNC in the PICU at RCH was modified for interhospital transport (Additional file 3). Medical and nursing transport staff were all trained for the HHFNC use in the PICU beforehand. The main indication included respiratory distress from bronchiolitis, pneumonia, etc. The initial setting included flow rate of two litter per kilogram per minute when patient’s body weight was up to 10 kg, and FiO_2_ of 0.4–0.5 with the target saturation of 90–98%. Closely monitoring patients for response was essential, and escalation of respiratory support should be considered if patients have not been stabilized with HHFNC. Details were summarized in the Additional file 3.

### Patient selection

We included all children who were admitted to the PICU at RCH with or without interhospital transport with the primary diagnosis of respiratory distress, or with associated diagnoses of prespecified respiratory distress in the study period (January 2010–December 2019). (Additional file 1).

Exclusion criteria included 18 years old or older, the primary diagnosis of sepsis/septic shock/cardiac disease/neurological disease/trauma/toxin/burn, cardiac arrest prior to transport team arrival or PICU admission, tracheostomy, children transported by other retrieval services than PIPER, elective PICU admission, previous PICU admissions within the same hospital admission, and PICU readmission within 24 h after PICU discharge.

### Outcomes

The primary outcome was the length of PICU stay.

Secondary outcomes were duration of respiratory support (defined as the duration of combined invasive and non-invasive ventilation use in PICU), prevalence of invasive ventilation in PICU, adverse events during transport (defined as escalation of respiratory support mode, cardiac arrest, need for resuscitation drugs), intubation within the first 4 h after PICU admission following interhospital transport, the length of hospital stay, and hospital mortality. The outcome follow-up was censored at 60 days to avoid the influence of extreme observations on outcomes.

### Additional variables

Patient-level variables and temporal variables related to institutional changes were collected from database and hospital protocols [10, 11]; patient characteristics, chronic conditions, cause of respiratory distress (asthma, bronchiolitis, croup, pneumonia, other respiratory diseases), transport data, and outcomes. Details were described in the Additional file 1. Age was categorized into 0– < 1, 1– < 2, 2– < 5, 5– < 18 years. For temporal variables, July 2011, April 2013, and January 2014 were included as HHFNC was introduced in PICU, emergency department, and ward, respectively (Additional file 2: Table S1).

### Statistical analysis

We used a comparative interrupted time series approach with the patient- and temporal covariate adjustment. A comparative interrupted time series analysis is a quasi-experimental design which can estimate the longitudinal outcome change by the intervention by comparing the outcome change in the intervention group over the outcome change in the comparative group between pre- and post-intervention eras. Compared to the interrupted time series analysis only including the intervention group, this comparative model allowed us to calculate a more robust estimate because the outcome trend change due to secular factors and temporal changes could be set off by subtracting the trend change in the comparative group from one in the intervention group [15]. Patients were divided in 1-year time period, and categorized in pre-intervention era (2010–2014) and post-intervention era (2015–2019). The data analysis was performed according to the prespecified plan as described in the Additional file 1.

First, we reviewed the patient characteristics between transported children and non-transport children to assess the comparability between the two cohorts [15–17]. Post-hoc sensitivity analysis was performed if there were marked differences in the patient characteristics between two cohorts. The interrupted time series analysis requires assumptions; (a) the trend is linear (or can be transformed to be linear), (b) the patient characteristics is consistent over time, (c) the intervention was introduced in a certain time [18]. Detailed reviews of these assumptions were summarized in the Additional file 2 (3. Assessment of model assumptions). In short, there were no evidences against using a comparative interrupted time series approach.

The cause of regression models for each outcome was chosen based on clinical knowledge from previous studies, the histogram for the distribution of actual and log-transformed outcomes, and model fitting of regression models by using the Akaike information criteria [19]. Consequently, a linear regression with the log-transformed outcome was chosen for the length of stay in PICU and hospital while a zero-inflated binomial negative regression was chosen for the duration of respiratory support as a substantial proportion of observed outcomes were zero (Additional file 2: 2. Model specification). Hence, study results were presented as the ratio or predicted absolute difference with 95% confidence interval (CI) according to the selected regression model type.

To evaluate the outcome effect by the intervention, the difference in level changes in transported and non-transport children between pre- and post-intervention era was analyzed by including an interaction between transported children and post-intervention era in the final model. This measurement provided the level change of the outcome between pre- and post-intervention era. We chose the level change rather than the trend change based on a specialists’ discussion that the effect of the implementation was likely to present soon given HHFNC use on interhospital transport had increased timely based on their clinical experiences, which was assured in the preliminary analysis of collected data. Details of final model specifications were described in the Additional file 2 (2. Model specification).

We performed a number of prespecified sensitivity analyses to assess the robustness of the final model. One of sensitivity analyses was a model excluding low severity score on PICU admission (Paediatric Index of Mortality (PIM)-2 score) [20]. This sensitivity analysis was highly informative because previous literature have reported an increased number of PICU admission with less severe respiratory distress after implementing HHFNC on the settings outside of PICU, which may violate one of assumptions of this study design (consistent patient characteristics over year) [7]. We also analyzed with a model adjusted for PIM-2 as an additional covariate. In addition, we performed a difference-in-differences approach with matched cohorts between transported and non-transport children developed by estimating the likelihood of transport and adjusting for the study covariates and admission year with a one-to-one nearest neighbor propensity score matching without replacement [21]. This sensitivity analysis is extremely beneficial because the comparison of matched cohorts did not require assumptions of a comparative interrupted time series analysis. We scheduled another sensitivity analysis with a model allowing the outcome effect by the intervention to vary each year in the post-intervention era to assess whether the effects of the intervention were consistent.

For the population-adjusted analysis, the annual sum of each respiratory support mode use was aggregated by the admission source. Then, the annual sum was adjusted for the pediatric population in 2015 by using the pediatric population for each year by referring the data from the Victoria by Australian Bureau of Statistics (Additional file 2: Table S8). The average of population-adjusted annual respiratory support uses was calculated by the era and admission source.

Mann–Whitney U test, chi-square test or Fisher’s exact test was used for other comparisons according to the characteristics of variables. Two-tailed *p* values < 0.05 were considered significant. STATA 14 (StataCorp LLC, College Station, TX, USA) was used for all statistical analyses.

## Results

### Patient demographics

**Fig. 1:**
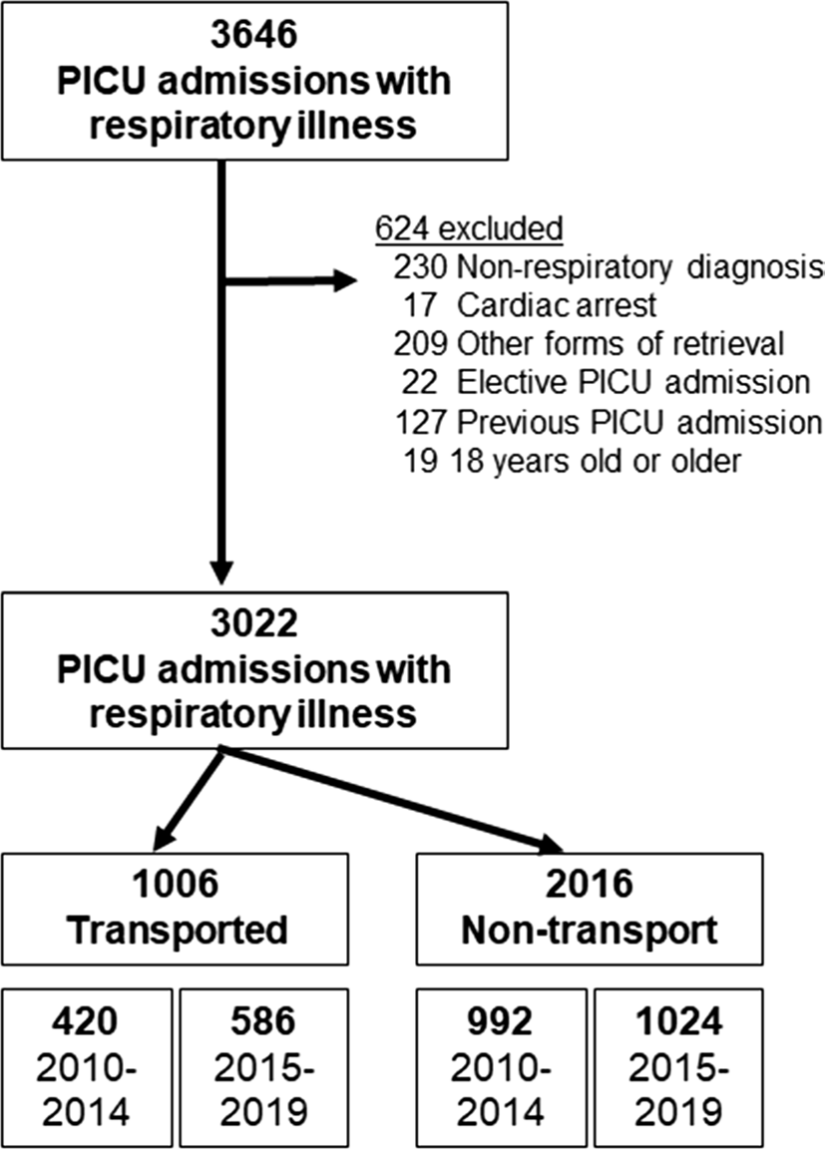
Study flow

**Fig. 2:**
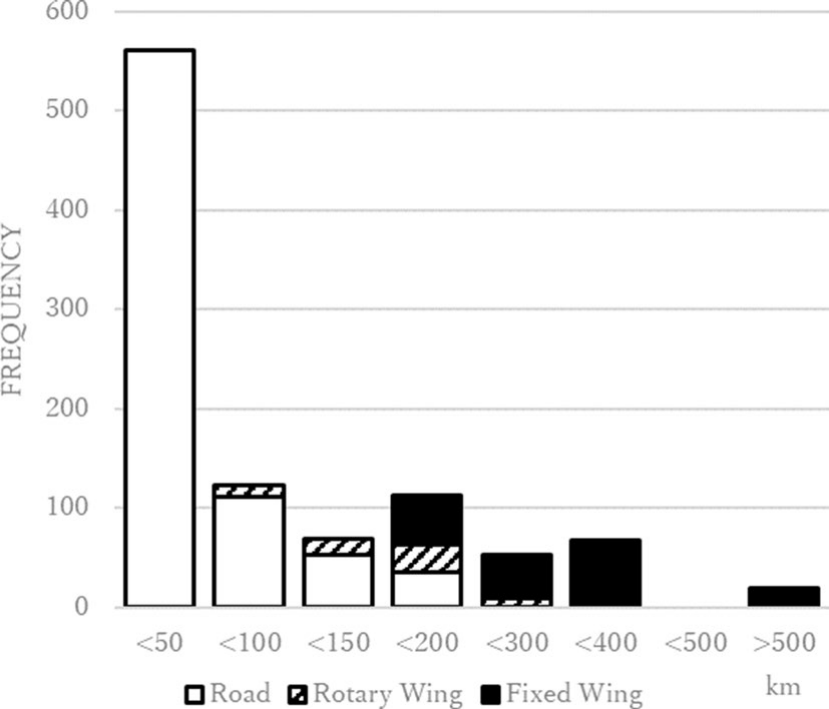
Number of retrievals by trip distance (*n* = 1006). Distribution of transport distance and retrieval mode in 1006 interhospital transports by a specialized paediatric retrieval service to a paediatric intensive care unit over 10 years

3022 children were included for the analysis after excluding 624 who met exclusion criteria (Fig. 1). In pre- and post-intervention era, there were 420 and 586 transported children while there were 992 and 1024 non-transport children. Median [interquartile range (IQR)] age was 1.4 (0.7–4.5) years. Pneumonia (39.1%) and bronchiolitis (34.3%) were common causes of respiratory distress, which were followed by asthma (12.9%) and croup (10.2%). The median (IQR) distance of the transport was 30.4 (17.6–150) km. The median (IQR) transport time from referral hospitals to RCH was 0.7 (0.5–1.7) h. Details of transport were summarized in the Fig. 2.

### Comparisons of patient characteristics

Comparisons of patient characteristics were summarized in the Table 1. In a comparison between pre- and post-intervention era in each cohort, there was no marked imbalance in the most of patient-level variables although there was an increasing trend in home-ventilation dependent and previous PICU admission in non-transport children. Although non-transport children were more likely to have underlying diseases such as chronic encephalopathy and previous PICU admission than transported children, overall other variables were not substantially unbalanced. Post-hoc sensitivity analysis with regard to these three covariates showed a similar study result as the primary test (Additional file 2: 5. Other considerations).

**Table 1:** Patient demographics

	Transported, *n* (%)		SD	Non-transport, *n* (%)		SD
	2010–2014 *n* = 420	2015–2019 *n* = 586		2010–2014 *n* = 992	2015–2019 *n* = 1024	
*Age*						
< 1 year	152 (36.2)	203 (34.6)	0.03	418 (42.1)	358 (35.0)	0.15
1–< 2 years	102 (24.3)	159 (27.1)	0.07	175 (17.6)	210 (20.5)	0.07
2–< 5 years	77 (18.3)	117 (20.0)	0.04	170 (17.1)	191 (18.7)	0.04
5–< 18 years	89 (21.2)	107 (18.3)	0.07	229 (23.1)	265 (25.9)	0.06
Male	266 (63.3)	350 (59.7)	0.07	593 (59.8)	613 (59.9)	0.00
*Respiratory category*						
Asthma	82 (19.5)	92 (15.7)	0.10	104 (10.5)	113 (11.0)	0.02
Bronchiolitis	145 (34.5)	177 (30.2)	0.09	372 (37.5)	343 (33.5)	0.08
Croup	59 (14.0)	99 (16.9)	0.08	78 (7.9)	71 (6.9)	0.04
Pneumonia	111 (26.4)	180 (30.7)	0.09	412 (41.5)	479 (46.8)	0.11
Others	23 (5.5)	38 (6.5)	0.04	26 (2.6)	18 (1.8)	0.06
Haemato-oncological disease	2 (0.5)	12 (2.0)	0.14	26 (2.6)	32 (3.1)	0.03
Neuromuscular disease	2 (0.5)	6 (1.0)	0.06	21 (2.1)	28 (2.7)	0.04
Airway disease	19 (4.5)	14 (2.4)	0.12	51 (5.1)	68 (6.6)	0.06
Lung disease	25 (6.0)	34 (5.8)	0.01	78 (7.9)	91 (8.9)	0.04
Chromosomal abnormality	22 (5.2)	21 (3.6)	0.08	67 (6.8)	52 (5.1)	0.07
Chronic encephalopathy	23 (5.5)	24 (4.1)	0.06	163 (16.4)	179 (17.5)	0.03
Cyanotic congenital cardiac disease	9 (2.1)	9 (1.5)	0.05	23 (2.3)	25 (2.4)	0.00
Prematurity	74 (17.6)	96 (16.4)	0.03	199 (20.1)	168 (16.4)	0.09
Home-ventilation dependent	0 (0.0)	7 (1.2)	0.16	28 (2.8)	72 (7.0)	0.20
Previous PICU admission	41 (9.8)	52 (8.9)	0.03	214 (21.6)	306 (29.9)	0.19
PIM-2*, median (IQR)*	0.7 (0.2–1.4)	0.6 (0.2–1.0)	0.05	0.7 (0.2–1.4)	0.8 (0.27–1.1)	0.05
Unadjusted PICU length of stay*, day, median (IQR)*	2.2 (1.1–4.2)	1.7 (0.9–3.2)		2.4 (1.3–4.9)	2.1 (1.2–4.5)	

*SD* standardized difference, *PIM* Paediatric Index of Mortality, *IQR* interquartile range, *PICU* paediatric intensive care unit

### Chronological trend of HHFNC

**Fig. 3:**
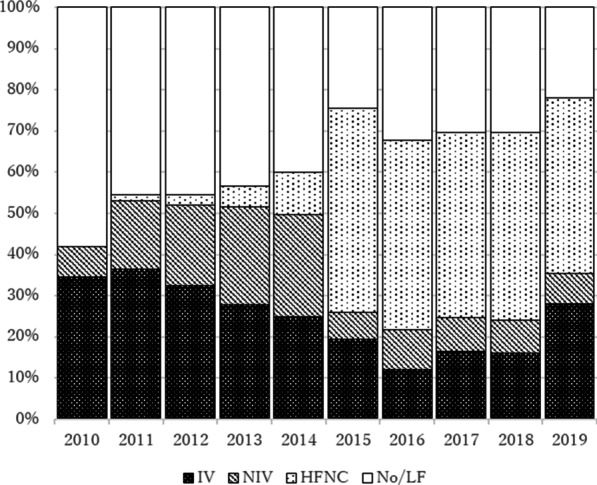
Percentage of retrieval by respiratory support type over year (*n* = 1006). *PICU* paediatric intensive care unit, *IV* invasive ventilation, *NIV* non-invasive ventilation, *HHFNC* humidified high-flow nasal cannula, *No/LF* no respiratory support or low-flow oxygen

On retrieval, HHFNC was used in 5.0% (21/420) and 45.9% (269/586) in pre- and post-intervention era. In PICU, HHFNC was used in 36.4% (153/420) and 60.1% (352/586) of transported children, 52.2% (518/992) and 70.8% (725/1024) of non-transport children, in pre- and post-intervention era respectively. In 2015, there were a sharp increase in the usage of HHFNC, and reduced utilization of invasive and non-invasive ventilation on transport (Fig. 3).

### Primary analysis

In an unadjusted model, median (IQR) PICU length of stay was 2.2 (1.1–4.2) days and 1.7 (0.9–3.2) days in the pre- and post-intervention era in transported children while the figure was 2.4 (1.3–4.9) and 2.1 (1.2–4.5) in non-transport children (Table 1).

**Fig. 4:**
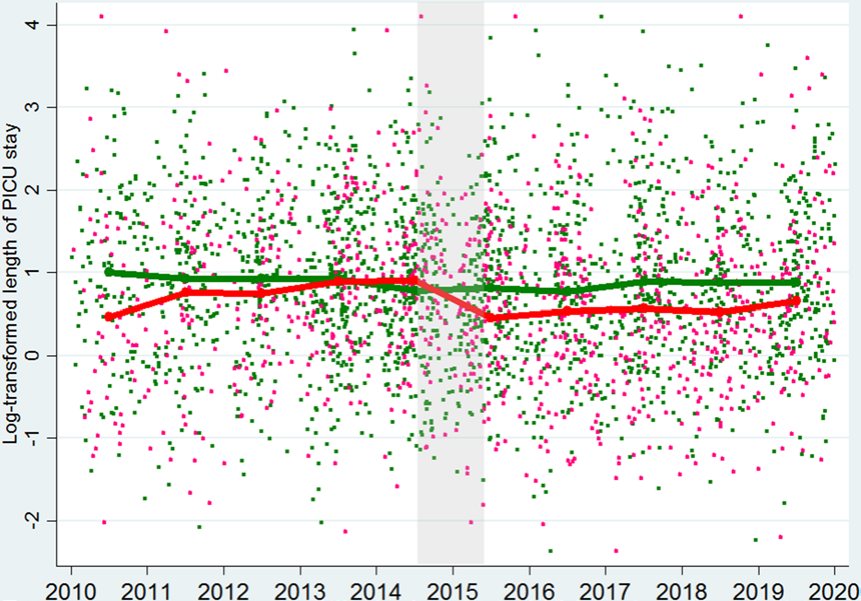
Distribution of observations and the fitted values based on the final model (*n* = 3022). *PICU* paediatric intensive care unit. The implementation of humidified high-flow nasal cannula on the interhospital transport service occurred around the beginning of 2015, which was highlighted by gray background. A red connected line presents the mean of fitted values of log-transformed PICU length of stay (day) in transported children with respiratory distress over year based on the final model while a green line presents the mean value in non-transport children with respiratory distress. Scattered dots present 3022 observations of the log-transformed PICU length of stay with 1006 pink dots being in transported and 2016 green ones being non-transport children. As *y*-axis presents the log-transformed PICU length of stay, 0, 1,2 in *y*-axis indicated around 1, 2.7, 7.4 days

The multivariable linear regression model showed that the intervention was associated with reduced PICU length of stay (ratio: 0.64, 95% confidence interval (CI) 0.49–0.83, *p *= 0.001). The predicted PICU length of stay decreased by − 9.1 (95% CI − 14.8 to − 3.3) hours in transported children based on the fitted final model while the figure increased by 1.5 (− 2.8 to 5.9) h in non-transport children. By comparing the changes in the two cohorts, the intervention was associated with a shortened PICU length of stay by − 10.6 (95% CI − 16.9 to − 4.3) h (Table 2). To describe the primary test result graphically, scatter plots for observed primary outcomes and mean of fitted values on the final model were illustrated in the Fig. 4, describing the reduction of the outcome around 2015 in transported children. The estimated outcome effect by the temporal variables were all not significant (Additional file 2: 3.5 No significant effects by temporal variables).

**Table 2:** The outcome effect by the implementation of humidified high-flow nasal canula on transport (n = 3022)

Outcome	Cohort	Level change between 2010–2014 and 2015–2019^a^	Difference in level changes between two cohorts^b^	*p*
*PICU length of stay, ratio (95% CI)*^*c*^				
	Transported	0.69 (0.54–0.87)	0.64 (0.49–0.83)	0.001
	Non-transport	1.07 (0.89–1.28)	–	
*Estimated PICU length of stay, absolute difference, hour (95% CI)*^*d*^				
	Transported	− 9.1 (− 14.8 to − 3.3)	− 10.6 (− 16.9 to − 4.3)	–
	Non-transport	1.5 (− 2.8 to 5.9)	–	
IV + NIV hours, *absolute difference, hour (95% CI)*^*e*^				
	Transported	− 17.0 (− 37.1 to 3.1)	− 25.1 (− 47.9 to − 2.3)	0.03
	Non-transport	8.1 (− 8.0 to 24.2)	–	
*IV hours, absolute difference, hour (95% CI)*^*e*^				
	Transported	− 11.5 (− 21.1 to − 1.9)	− 22.4 (− 33.8 to − 10.9)	< 0.001
	Non-transport	10.9 (-2.0 to 19.7)	–	

–, not applicable

*PICU* paediatric intensive care unit, *CI* confidence interval, *IV* invasive ventilation, *NIV* non-invasive ventilation

^a^Level change between the pre-intervention era (2010–2014) and post-intervention era (2015–2019)

^b^This is the main result of the study, presenting the outcome effect by the intervention by subtracting the outcome level changes between pre- and post-intervention era in non-transport children from the outcome level change in transported children

^c^The ratio was estimated based on a linear regression model with the log-transformed outcome

^d^The absolute difference of PICU length of stay between pre- and post-intervention era was predicted by the fitted linear regression model with the covariate adjustment

^e^The absolute difference of the outcome was predicted based on a zero-inflated binomial regression as a substantial proportion of observed outcomes were zero

### Propensity score-matched cohorts and other sensitivity analyses

By the propensity score matching between transported and non-transport children, 989 pairs of children were selected whose characteristics were balanced (Additional file 2: Table S7). The difference-in-differences approach with matched cohorts showed the estimated outcome effect with a ratio of 0.78 (95% CI 0.66–0.92, *p *= 0.004) (Table 3). Outcome effects were consistent across all sensitivity analyses in the Table 3. The model excluding children with low severity score showed the estimated effect with a ratio of 0.73 (95% CI 0.53–1.00, *p *= 0.049). Another sensitivity analysis using a model allowing outcome effects to differ each year also showed a consistent result (Additional file 2: Table S6).

**Table 3:** Sensitivity analysis for the primary test

	Analysis method	*n*	Estimated effect		*p*
			Ratio	(95% CI)	
	Final model	3022	0.64	(0.49–0.84)	0.001
a	Bandwidth 80% (including 2011–2018)	2554	0.66	(0.49–0.88)	0.01
b	Bandwidth 60% (including 2012–2017)	2046	0.74	(0.52–1.04)	0.08
c	Washout-period (excluding 2014–2015)	2253	0.53	(0.34–0.82)	0.004
d	120 monthly time periods	3022	0.67	(0.52–0.86)	0.002
e	Consistent severity (excluding low PIM-2)	2274	0.73	(0.53–1.00)	0.049
f	Additional adjustment for severity score (PIM-2)	3022	0.66	(0.51–0.86)	0.002
g	Model (using matched cohort)	1978	0.78	(0.66–0.92)	0.004
h	Model (using discontinuity regression for transport)	1006	0.70	(0.51–0.95)	0.02
i	Uncensored outcome	3022	0.65	(0.50–0.84)	0.001
j	Excluding extremely influential observations	2787	0.66	(0.53–0.83)	< 0.001

*CI* confidence interval, *PIM* paediatric index of mortality

The sensitivity analysis was performed to assess the robustness and resistance of the final model which was designed to obtained the estimated effect by the implementation of humified high-flow nasal cannula on interhospital transport on the primary outcome (length of stay in the intensive care unit)

In the final model, a multivariable linear regression with the log-transformed outcome was used. The study period was Jan2010-Dec2019

^ab^In these analyses, children were included for the shorter study duration than the original 10-year study period

^c^In this analysis, admissions from Jan2014-Dec2015 were excluded

^d^In this analysis, instead of the yearly time period, the monthly time period was used with indicator variables for each month

^e^In this analysis, children with 25 percentile or lower severity score were excluded

^f^In this analysis, the model was expanded with an additional severity score

^g^In this analysis, matched cohorts between transported and non-transport children were generated and then the difference in differences between pre- and post-intervention era in two cohorts were calculated

^h^In this analysis, only transported children were included while the model was same as the final model

^i^In this analysis, the uncensored outcome was used

^j^In this analysis, we excluded 235 extremely influential observations based on the Difference in Fits (DIFFITS)

### Analysis of respiratory support use

The estimated reduction of combined invasive and non-invasive ventilation use by the intervention was − 25.1 h (95% CI − 47.9 to − 2.3 h, *p *= 0.03) based on a zero-inflated negative binomial regression with the covariate adjustment while the estimated reduction in invasive ventilation solo was − 22.4 h (95% CI − 33.8 to − 10.9 h, *p *< 0.001). There was a lower prevalence of invasive ventilation use in the post-era among transported children (24% (141/586) vs. 38% (159/420), *p *< 0.001).

**Fig. 5:**
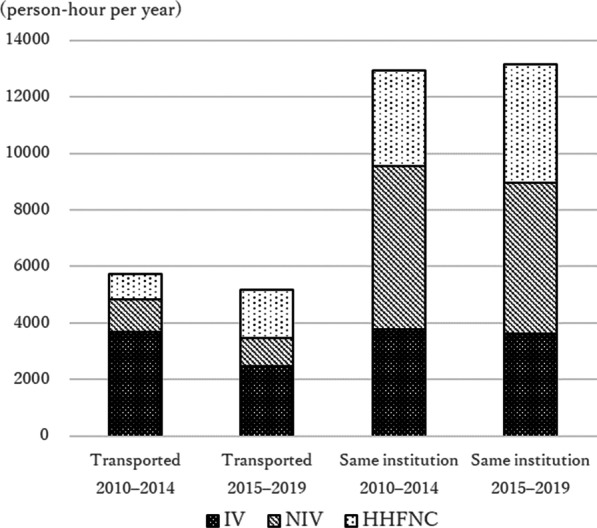
Population-adjusted duration of respiratory support use. *IV* invasive ventilation, *NIV* non-invasive ventilation, *HHFNC* humidified high-flow nasal cannula. After the total duration of each respiratory support use for each year was adjusted for the Victorian pediatric population in 2015, adjusted annual use of respiratory support were aggregated by the study era and admission source

In the population-adjusted analysis, among transported children, combined invasive and non-invasive ventilation use was 4837 and 3477 (person-hour per year) in pre- and post-era with 1360 reduction while HHFNC use was 906 and 1702 (person-hour per year) with 796 increase, resulting in the 564 reduction in the total duration of three modes of respiratory support. On the other hand, among non-transport children the reduction of combined invasive and non-invasive ventilation use was 593 (from 9553 to 8959) person-hour per year while increase of HHFNC use was 803 (from 3390 to 4193), resulting in 210 increase in three modes of respiratory supports (Fig. 5).

### Other results

Hospital mortality did not differ significantly. Among transported children seven (1.7%) and six (1.0%) died in pre- and post-era (*p *= 0.37). The duration of hospital stay decreased with a ratio of 0.65 (95% CI 0.52–0.82, *p *< 0.001) based on a multivariable linear regression model.

During interhospital transport, there was one intravenous adrenaline bolus for hypotension on an intubated child in the post-intervention era, while no escalation of respiratory support mode, and no cardiac arrest were observed. The intubation in PICU within 4 h after interhospital transport occurred in 3.1% (13/420) and 1.9% (11/586) in pre- and post-intervention era (Additional file 2: Table S9).

In the post-intervention era, 8.6% (23/269) of children transported with HHFNC were subsequently intubated during PICU stay with the median (IQR) time from admission to intubation of 19.6 (3.8–35.3) hours. The median (IQR) of the length of PICU stay was 5.9 (3.0–9.9) days in 23 children who failed with HFNC and intubated compared to 3.7 (2.4–6.7) days in 105 children directly intubated in referral hospitals in the post-intervention era. The hospital mortality did not differ very much (4.4% (1/23) vs. 2.9% (3/105)).

## Discussion

The main findings of this study were the implementation of HHFNC on interhospital transport was associated with (1) the reduction in the PICU length of stay, (2) the reduction in the duration of invasive and non-invasive ventilation use and hospital stay, and (3) the safety of HHFNC use on retrieval. This study has unique features; (a) a novel study to investigate the clinical impact of the implementation of HHFNC on transport, and (b) including broad respiratory distress and age groups with a comparison to many studies of HHFNC only including infants with bronchiolitis.

A quasi-experimental design with a comparative interrupted time series approach allowed us to estimate the outcome effect by the intervention. As a nature of the study design, the key critique is that the estimated clinical effect by the implementation of HHFNC may be overestimated by the potential effect of increased PICU admissions with less severe respiratory distress after implementing HHFNC on transport. As similar to previous studies [7], in this study the number of admissions increased in post-intervention era, the possibility of the overestimated effect in the primary test cannot be wiped out. Though, a number of sensitivity analyses including one with a severity cut-off showed consistent outcome effects, which assured that the estimated effect in this study could be the best available approximate of the outcome effect by the intervention.

This study demonstrated reduced PICU length of stay by the implementation of HHFNC on a transport team but the reason behind this association is not well understood. A possible speculation is that early use of HHFNC could be preventive of the escalation of respiratory supports [22]. Schibler et al. reported the reduced rate of intubation in infants with bronchiolitis by the HHFNC use in a PICU, but did not demonstrate a reduction in PICU length of stay [12]. No randomized controlled trials have demonstrated a reduction in the length of PICU stay and PICU admission rate by the early use of HHFNC in emergency departments or wards, while there was a reduced rate of the treatment failure [22, 23]. Thus, there is a discrepancy between our result and previous trials. A plausible explanation is that randomized controlled trials might have failed to capture the effect of early use of HHFNC on respiratory distress by including a less severe cohort with the PICU admission rate being around 10% [22]. In addition, in many centres HHFNC was not provided in a non-PICU setting until recently, with increasing need for PICU admission and increasing PICU length of stay. A predictive method to identify children who would evolve severe respiratory distress might be useful to enable a future randomized controlled trial including high-risk children like adult trials [24], which would assure the effect we found of early use of HHFNC.

Another possible explanation is that intubation prior to transport among children with borderline respiratory distress could be reduced by HHFNC use on interhospital transport. Millan et al. reported that around 20% of children on HHFNC or non-invasive ventilation for respiratory failure were intubated for the transport and most of them were extubated shortly after transport, suggesting that there were a proportion of patients intubated purely for the transport [25]. Schlapbach et al. reported the reduced rate of children transported on invasive ventilation after implementing HHFNC on their retrieval service [7]. Similarly, in our study there was a sharp reduction of invasive ventilation use on transport. This is of great importance, considering the risk of intubation-associated adverse events outside of PICU [26]. Thus, HHFNC could be a safe and beneficial alternative to invasive ventilation in selected cohorts in a condition where close monitoring for the response and escalation of respiratory support mode are available. This concept highlights the knowledge gap that we need more evidence with regard to the indication of intubation versus HHFNC use for transport in the modern era in which relatively new modes of respiratory supports are available.

For safety considerations, monitoring the adverse events associated with the use of HHFNC is important since delaying intubation could lead to worse outcomes especially in children spontaneously breathing with high respiratory drive which could lead to lung injury [29]. Morris et al. showed that children who failed with HHFNC had increased mortality and prolonged length of PICU stay [30]. In our study the adverse event during transport was rare like previous studies [14], and there were no marked outcome differences between children directly intubated in referral hospitals and those who failed with HHFNC. However, our findings did not guarantee the safety of HHFNC use in different settings such as geographical locations that do not have a centralized transport team. In this sense, monitoring clinical outcomes associated with respiratory support during transport is valued, which could also lead to identifying the timing of intubation in transported children with evolving respiratory distress.

## Limitations

There were several limitations in this study. First, we tried to minimize the influence on the estimated effect by potential confounders by using many measurements (comparative group, restriction, adjustment, and sensitivity analysis). Though, there was still a possibility of residual confounding factors as a nature of the observational study. For example, as we included children for 10-year, other treatments for respiratory distress than respiratory support might have changed like antimicrobial drug, sedation, nutrition, physiotherapy etc. Second, due to the exclusion of children with sepsis, trauma, neurologic and cardiac compromise with secondary respiratory distress, this study result may not be able to be extrapolated to children with respiratory distress secondary to aforementioned etiologies. Third, the generalizability of findings in this study may be limited due to the difference in regional transport systems and transport distance. Forth, the importance of the primary outcome may vary depending on individual views [31]. Although the PICU length of stay was deemed important considering the social and economic circumstances among included children, this has not been studied well. Fifth, HHFNC-associated adverse effects were not fully assessed in this study such as nasal trauma, air leak, and need for additional sedation due to the limitation of available data.

## Conclusions

The implementation of HHFNC on interhospital transport was associated with reduced PICU length of stay and respiratory support use in PICU admissions with respiratory distress.

## Supplementary Information

**Additional file 1**. Analysis plan

**Additional file 2.** Supplementary online content.

**Additional file 3.** HHFNC protocol on transport.

## Data Availability

Not applicable.

## Abbreviations

PICU: Paediatric intensive care unit
HHFNC: Humidified high-flow nasal cannula
IQR: Interquartile range
CI: Confidence interval
