# Supplementary material for: Clinical impact of implementing humidified high-flow nasal cannula on interhospital transport among children admitted to a PICU with respiratory distress: a cohort study
Source: Crit Care. 2021 Jun 6;25:194. doi: 10.1186/s13054-021-03620-7 (PMC8180008; doi:10.1186/s13054-021-03620-7)
Supplement: Supplementary file 1 — Additional file 1. Analysis plan [file 13054_2021_3620_MOESM1_ESM.pdf]

Additional file 1 – Analysis plan

1. Study overview

This is a single-center retrospective cohort study to estimate the clinical impact of the implementation of humidified high-flow nasal cannula (HHFNC) on a specialized pediatric retrieval team which was put in practice late-2014 to 2015. Children admitted to a paediatric intensive care unit (PICU) with respiratory illness from 2010 to 2019 will be included.

2. Background

Critically-ill children with respiratory illness is an important population in terms of PICU admission number and healthcare resource consumption.[1234] During the retrieval of these children, several respiratory supports have been used by transport teams. HHFNC is a relatively new mode of non-invasive respiratory support which has been widely used for children with respiratory illness in Australia since around 2010s in intensive care, emergency department, ward and transport.[567] Regardless of the increasing use of HHFNC on transport worldwide, its clinical effects has not been known well. A study has reported that the use of HHFNC during interhospital transport was safe and associated with the reduced rate of invasive ventilation during transport.[8] Although it could be hypothesized that HHFNC use during transport could lead to improved patient outcomes and healthcare resource consumption (the length of PICU stay, the length of respiratory support use), these data are lacking. This is especially important when considering (i) the social situation that children and family are restricted in the tertiary hospital away from home, (ii) the substantial number of critically-ill children with respiratory illness worldwide, and (iii) planning for the quality improvement of transport system globally.

3. Study hypotheses

3.1 The use of HHFNC on transport may abolish the need for intubation prior to interhospital transport in children with respiratory illness who have borderline respiratory distress.

3.2 Early commencement of HHFNC can be therapeutic for some of respiratory illness (bronchiolitis, asthma, pneumonia), and prevent the escalation of respiratory supports and PICU admissions.

4. Objectives

The aim of this study is (1) to investigate if the implementation of HHFNC during interhospital transport reduces the length of PICU stay, (2) to assess the safety of HHFNC use during transport.

5. Analytical framework

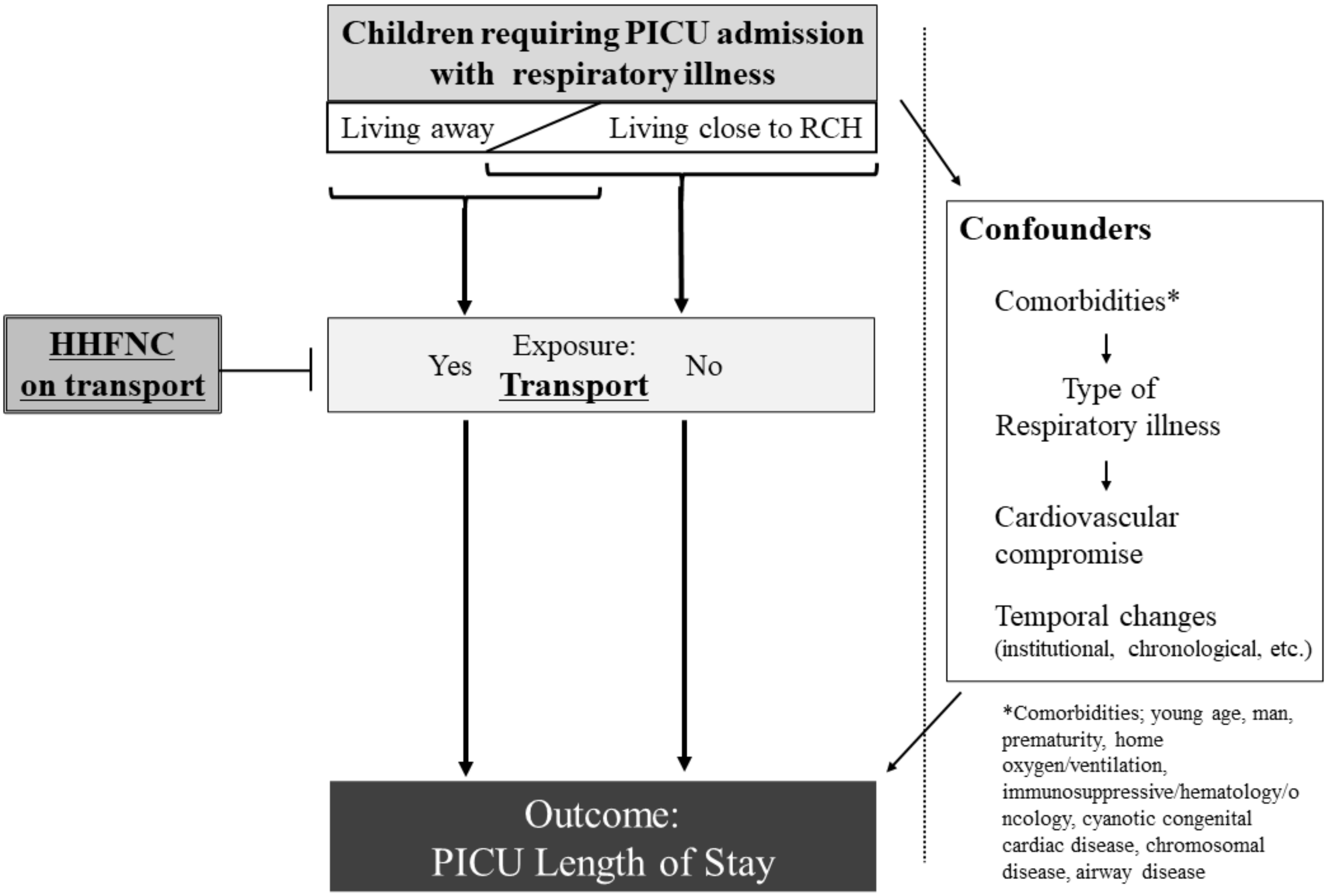

This analytical framework describes the key concept behind the development of the study protocol. As the principal factor to decide if children requiring PICU admission undergo interhospital transport is the location of children's residence, we assumed that two groups (those living away from RCH and those living close to RCH) could be basically comparable.

Transported children are exposed to the risk of the need for intubation prior to transport, delayed treatment of intensive care, and adverse events during transport compared to children admitted from the same institution.

We planned to adjust the outcome effect for cofounders in the final model. We planned to exclude children with a primary diagnosis of cardiovascular compromise because cardiovascularly-compromised children are heterogenous to the cohort of interest in this study in terms of respiratory management.

6. Methods

6.1 Study design

A single-center retrospective cohort study with a comparative interrupted time series approach

6.2 Setting

The Paediatric Infant Perinatal Emergency Retrieval (PIPER) is a specialized pediatric retrieval team who is responsible for all interhospital transport of critically-ill children < 18 years old in Victoria. In Victoria, all critically-ill children were transferred to one of two tertiary PICUs at the Royal Children's Hospital (RCH), Melbourne, and Monash Medical Center (MMC), Clayton. The destination has been decided based on the preset catchment. The admission number with PIPER retrieval for respiratory illness has been comparable between two hospitals. Basically, referral hospitals start to communicate with PIPER in an early stage of the escalation respiratory support (e.f., ongoing respiratory distress on low-flow oxygen therapy, HHFNC, and continuous positive airway pressure). All intubated children should have been transported to one of two tertiary PICUs.

The PICU at RCH is a 30-bed combined medical-surgical unit that accommodates approximately 1800 admissions annually. HHFNC was introduced in PIPER in the end of 2014 and actively used since 2015. At RCH, HHFNC was introduced in PICU in mid-2011, emergency department in April 2013, and ward in late-2013 to early-2014.[56]

6.3 Eligible patients

6.3.1 Inclusion criteria

We will include all children who were admitted to the PICU from interhospital transport or the same institution with the primary diagnosis of respiratory illness, or with associated diagnoses of prespecified respiratory illness* in the study period (January 2010 to December 2019).

*=bronchiolitis, upper respiratory infection, respiratory failure, pneumonia/pneumonitis, asthma, laryngotracheobronchitis/croup, epiglottitis, tracheitis, pertussis, lower respiratory infection, air leak, upper airway obstruction, pertussis, aponea, empyema, and foreign body

6.3.2 Exclusion criteria

Exclusion criteria includes 18 years old or older, the primary diagnosis of sepsis/septic shock/cardiac illness/neurological illness/trauma/toxin/burn, cardiac arrest prior to transport team arrival or PICU admission, children transported by other retrieval services than PIPER, elective PICU admission, previous PICU admissions within the same hospital admission, and PICU admission within 24 hours after PICU discharge.

6.4 Outcome

6.4.1 Primary outcome:

- The length of PICU stay

6.4.2 Secondary outcomes:

- Duration of respiratory support*
- Adverse events during transport (escalation of respiratory support, cardiac arrest, need for resuscitation drug)

- Hospital mortality
- Hospital length of stay
- The prevalence of invasive ventilation during PICU admission
- Intubation within the first four hours after PICU admission following interhospital transport

  *respiratory support will include invasive ventilation and noninvasive positive-pressure ventilation

6.5 Additional variables

Patient-level variables and temporal variables for institutional changes will be collected from database, hospital protocols[56]; patient characteristics and chronic conditions (age, sex, haemato-oncological disease, neuromuscular disease, airway disease, lung disease, chromosomal abnormality, chronic encephalopathy, cyanotic congenital cardiac disease, prematurity, home-ventilation dependent, previous PICU admission, pediatric index of mortality (PIM)-2 score[9]), respiratory illness type (asthma, bronchiolitis, croup, pneumonia, other respiratory illness), transport data (date of transport, referral hospital, destination of transport, respiratory support type before and during transport, adverse events during transport, PIPER's time staying at referral hospital, total trip time from retrieval base to PICU), outcomes (length of PICU stay, length of hospital stay, duration of each mode of respiratory support use during PICU stay, highest respiratory support, hospital mortality). Age will be categorized into <0.5, 0.5–<1, 1–<2, 2–<5, 5–<18 years based on discussions by specialists. The type of respiratory illness diagnosed by PIPER will be prioritized in the case there are conflicting diagnoses between PIPER and PICU database. The outcome follow-up will be censored at 60 days to avoid the influence of extreme observations on outcomes.

For temporal variables, July 2011, April 2013, and January 2014 will be included as HHFNC was introduced in PICU, emergency department, and ward, respectively. January 2012 will be included as the PICU was expanded to 30 beds.

7. Primary analysis

We will use a comparative interrupted time series approach with the patient- and temporal covariate adjustment. A comparative interrupted time series analysis is a quasi-experimental design which can estimate the longitudinal outcome change by the intervention by comparing the outcome change in the intervention group over the outcome change in the comparative group between pre- and post-intervention periods. We considered the possibility that temporal and institutional changes (the implementation of HHFNC on PICU/emergency department/pediatric ward, and increase in the PICU bed number in 2012) may influence outcomes over year. Compared to the interrupted time series analysis only using the intervention group, this comparative model will allow us to calculate a more robust estimate because the outcome trend change due to secular factors and temporal changes could be set off by subtracting the trend change in the comparative group from one in the intervention group.

The model specification is as below;

$$Y = \beta_0 + \beta_1 *transport + \beta_2 *year + \beta_3 *year*transport + \beta_4 *intervention* transport + \beta_5 *intervention + \beta_6 *intervention*year*transport + \beta_7 *intervention*year + \sum_{v=1}^{V} \lambda v X v + \varepsilon$$

Y=the outcome of interest; transport = 1 in transported children, 0 in children from the same institute; year=centralized time as a continuous variable (i.e. calendar year - 2015); intervention = 1 in post-intervention period (2015–2019), 0 in pre-intervention period (2010–2014); λ=coefficient of covariates; X= study covariates.

The type of the final regression model for the primary outcome will be selected among a linear regression with the actual or log-transformed outcome, a Poisson regression, and a negative binomial regression based on the distribution of observed outcomes, and the model fitting using the Akaike information criteria. In other outcomes which will contain a proportion of zero such as duration of respiratory support, a zero-inflated binomial regression or a zero-inflated Poisson regression will be used.

The study covariates were selected based on clinical knowledge and previous publications; age, sex, type of respiratory illness, haemato-oncological disease, neuromuscular disease, airway disease, lung disease, prematurity, cyanotic congenital cardiac disease, chromosomal abnormality, chronic encephalopathy, prematurity, home-ventilation dependent, previous PICU admission, and temporal variables (HHFNC use in PICU, emergency department, ward, and increase in the PICU bed number). After running models with several sets of covariates, the confounders to be included in the final model will be selected based on the model fitting using the Akaike information criteria, and from clinical viewpoints.

Prior to the primary test, four components (homogenous comparative group, linear trend, constant composition, timely enforcement of the intervention) will be reviewed to ascertain that the comparative interrupted time series approach is viable in the study cohort.[10111213]

To simplify the model, a difference-in-differences analysis will be used if there are similar trends between transported children and children from the same institution. This assumption will be assessed by the difference of trends between two (i.e. $\beta 3$ in the aforementioned model including children in the pre-intervention era).

8. Other consideration

We will perform the primary test without seasonal variables because included children were divided in one-year time period. Considering the characteristics of the time series analysis, we will assess the effect of

seasonality by expanding the final model with indicator variables for each month so as to evaluate if seasonal variables should be included in the final model.

We will perform post-hoc sensitivity analyses if the characteristics of collected data varies substantially by time or by the exposure to the transport.

9. Sensitivity analysis

We will perform a number of sensitivity analyses to examine the robustness and resistance of the primary test. (i) A model excluding low severity score on PICU admission based on Paediatric Index of Mortality (PIM)-2 score was planned. This sensitivity analysis will be highly informative because previous literature have reported an increased number of PICU admissions with less severe respiratory illness after implementing HHFNC on the settings outside of PICU, which may violate one of assumptions of this study design (consistent patient characteristics over year). (ii) We also scheduled a difference-in-differences approach with a matched cohort between transported children and those from the same institution developed by a nearest neighbor propensity score matching without replacement using transport as exposure, adjusting for the study covariates and admission year. This sensitivity analysis is extremely beneficial because the comparison of the matched cohort does not require assumptions of a comparative interrupted time series analysis. Other analyses include different bandwidth including (iii) eight years (2011 to 2018) (iv) six years (2012 to 2017), (v) excluding 2014 and 2015 as a wash-out period, (vi) using one month time period rather than one year time period, (vii) discontinuity regression only including transported children, (viii) using indicator variables for each year to measure the outcome effect by the intervention rather than using the level and trend change, (ix) additional adjustment for severity score on admission, and (x) excluding extreme observations by the Difference in Fits (DIFFTS).

9 Population-adjusted analysis for respiratory support use

First the total duration of each mode of respiratory support use will be calculated by the cohort. Then, the annual sum was adjusted for the pediatric population in 2015 by using the pediatric population data from the Victoria by Australian Bureau of Statistics. The adjusted respiratory support use will be aggregated in the pre-intervention era and post-intervention era, respectively.

References

1. Gregory CJ, Nasrollahzadeh F, Dharmar M, Parsapour K, Marcin JP. Comparison of critically ill and injured children transferred from referring hospitals versus in-house admissions. *Pediatrics*. 2008;121(4). doi:10.1542/peds.2007-2089
2. Moynihan K, McSharry B, Reed P, Buckley D. Impact of Retrieval, Distance Traveled, and Referral Center on Outcomes in Unplanned Admissions to a National PICU. *Pediatr Crit Care Med*. 2016;17(2):e34-e42. doi:10.1097/PCC.0000000000000586
3. Schlapbach LJ, Straney L, Gelbart B, et al. Burden of disease and change in practice in critically ill infants with bronchiolitis. *Eur Respir J*. 2017;49(6). doi:10.1183/13993003.01648-2016
4. Ramnarayan P, Thiru K, Parslow RC, Harrison DA, Draper ES, Rowan KM. Effect of specialist retrieval teams on outcomes in children admitted to paediatric intensive care units in England and Wales: A retrospective cohort study. *Lancet*. 2010;376(9742):698-704. doi:10.1016/S0140-6736(10)61113-0
5. Ten Brink F, Duke T, Evans J. High-flow nasal prong oxygen therapy or nasopharyngeal continuous positive airway pressure for children with moderate-to-severe respiratory distress? *Pediatr Crit Care Med*. 2013;14(7):e326-e331. doi:10.1097/PCC.0b013e31828a894d
6. Long E, Babl FE, Duke T. Is there a role for humidified heated high-flow nasal cannula therapy in paediatric emergency departments? *Emerg Med J*. 2016;33(6):386-389. doi:10.1136/emermed-2015-204914
7. Schibler A, Pham TMT, Dunster KR, et al. Reduced intubation rates for infants after introduction of high-flow nasal prong oxygen delivery. *Intensive Care Med*. 2011;37(5):847-852. doi:10.1007/s00134-011-2177-5
8. Schlapbach LJ, Schaefer J, Brady AM, Mayfield S, Schibler A. High-flow nasal cannula (HFNC) support in interhospital transport of critically ill children. *Intensive Care Med*. 2014;40(4):592-599. doi:10.1007/s00134-014-3226-7
9. Slater A, Shann F, Pearson G. PIM2: A revised version of the Paediatric Index of Mortality. *Intensive Care Med*. 2003;29(2):278-285. doi:10.1007/s00134-002-1601-2
10. Kontopantelis E, Doran T, Springate DA, Buchan I, Reeves D. Regression based quasi-experimental approach when randomisation is not an option: Interrupted time series analysis. *BMJ*. 2015;350:1-4. doi:10.1136/bmj.h2750
11. Gigli KH, Davis BS, Yabes JG, et al. Pediatric outcomes after regulatory mandates for sepsis care. *Pediatrics*. 2020;146(1). doi:10.1542/peds.2019-3353
12. Kahn JM, Davis BS, Yabes JG, et al. Association Between State-Mandated Protocolized Sepsis Care and In-hospital Mortality Among Adults With Sepsis. *JAMA - J Am Med Assoc*. 2019;322(3):240-250. doi:10.1001/jama.2019.9021

13. Austin PC. Balance diagnostics for comparing the distribution of baseline covariates between treatment groups in propensity-score matched samples. *Stat Med*. 2009;28(25):3083-3107.
