# Supplementary material for: Clinical impact of implementing humidified high-flow nasal cannula on interhospital transport among children admitted to a PICU with respiratory distress: a cohort study
Source: Crit Care. 2021 Jun 6;25:194. doi: 10.1186/s13054-021-03620-7 (PMC8180008; doi:10.1186/s13054-021-03620-7)
Supplement: Supplementary file 2 — Additional file 2. Supplementary online content. [file 13054_2021_3620_MOESM2_ESM.pdf]

Additional file 2 – Supplementary online content

Supplementary Methods

1 Deviations from the prespecified analysis plan
2 Model specification
3 Assessment of model assumptions
4 Confounders
5 Other considerations

Supplementary Tables

Supplemental Table 1 Full list of variables and definitions
Supplemental Table 2 Comparison of patient characteristics by the source of admission
Supplemental Table 3 Regression model fitting
Supplemental Table 4 Models with several set of covariates
Supplemental Table 5 Trend changes of the length of stay in the intensive care unit
Supplemental Table 6 Sensitivity analysis with indicator variable for each year post intervention
Supplemental Table 7 Characteristics of propensity-score matched cohorts
Supplemental Table 8 Pediatric population in Victoria state
Supplemental Table 9 The intubation after PICU admission following interhospital transport

Supplementary Figures

Supplemental Figure 1 Distribution of observed outcomes
Supplemental Figure 2 Distribution of log-transformed observed outcomes
Supplemental Figure 3 Trend of the observed outcome over year by the source of admission
Supplemental Figure 4 Percentage of humidified high flow nasal cannula use during interhospital transport
Supplemental Figure 5 Regression diagnostics
Supplemental Figure 6 Observed outcomes and fitted values based on the final model by admission source
Supplemental Figure 7 Percentage of transported children with respiratory distress by the destination

1 Deviations from the prespecified analysis plan

1.1 Age category

To simplify the final model, the age category was modified. As children aged <6 month old and those aged 6–<12 month old have similar patient-level characteristics, these age groups were combined into a age category of 0–<1 year old.

1.2 Additional outcome report

As one of secondary outcomes, we decided to add the duration of invasive ventilation so as to provide more detail results.

1.3 Additional sensitivity analysis

In the comparison of patient characteristics between transported children and non-transport children, three underlying conditions disproportionately distributed (chronic encephalopathy, home-ventilation dependent, and previous paediatric intensive care unit (PICU) admission). Post-hoc analyses with regard to these covariates were performed. A sensitivity analysis using uncensored outcomes were also added to confirm that the preset censoring did not distort the result of the primary test.

2 Model specification

To estimate the outcome effect by the intervention of the study, we used a comparative interrupted time series (CITS) analysis as prespecified in the protocol. Compared to the interrupted time series analysis only using the intervention group, this comparative model allowed us to calculate a more robust estimate because the outcome trend change due to secular factors and temporal changes could be set off by subtracting the outcome trend change in the comparative group from one in the intervention group. Patients were divided in one-year time period, and categorized into pre-intervention era (2010–2014) and post-intervention era (2015–2019) as per protocol. The model included a time variable, exposure to the interhospital transport, post-intervention era, and interactions among these. The trends were allowed to differ in the post-intervention era. This model also controlled for patient-level variables and temporal variables.

This model is specified as:

$$Log\ Y = \beta_0 + \beta_1 * transport + \beta_2 * year + \beta_3 * year * transport + \beta_4 * intervention * transport + \beta_5 * intervention + \beta_6 * intervention * year * transport + \beta_7 * intervention * year + \sum_{v=1}^{V} \lambda v X v + \varepsilon$$

Y=length of PICU stay (day); transport = 1 in transported children, 0 in non-transport children; year=centralized admission year as a continuous variable (i.e. calendar year - 2015); intervention = 1 in post-intervention period (2015–2019), 0 in pre-intervention era (2010–2014); λ=coefficient of covariates; X= study covariates (age, sex, cause of respiratory distress, haemato-oncological disease, neuromuscular

disease, airway disease, lung disease, chromosomal abnormality, chronic encephalopathy, prematurity, home-ventilation dependent, previous PICU admission, and temporal variables (HHFNC use in PICU, emergency department, and ward))
In this model, the point estimate of $\beta 4$ was interpreted as the outcome effect by the intervention. We used the same standard deviation in the outcome of interest for both transported and non-transport children. The plausibility of the same standard deviation was examined in the regression diagnostics. A multivariable linear regression model with a log-transformed outcome was used according to the preset selection method for regression models based on the distribution of observed outcomes and model fitting (Supplemental figure 2, 3 and table 3). Following components with regard to the model assumption of the CITS analysis were reviewed to ascertain that the CITS analysis is a viable approach in the study cohort. We treated all admissions as independent observations since we assumed that the outcome effect would not be distorted much considering the limited number of PICU readmission within a short period. This assumption was examined by the model including only first PICU admission in the study period. We did not use a difference-in-differences approach for the primary test as the trends in the pre-intervention era varied significantly between the two cohorts.

3 Assessment of model assumptions

3.1 Homogenous comparative group

To examine whether transported and non-transport children are comparative enough to be included in the CITS analysis, patient-level characteristics which could be potential confounders were compared by a descriptive data analysis as prespecified. (Supplemental table 2) Although non-transport children were more likely to have underlying diseases such as chronic encephalopathy and previous PICU admission than transported children, overall other variables were comparable. Hence, we considered that there was no major heterogeneity suggesting against using the CITS approach. The post-hoc analysis with regard to these covariates (chronic encephalopathy, home-ventilation dependent, and previous PICU admission) was added.

3.2 Linear trend

Based on literature review, the chronological trend of the length of PICU stay was linear (or can be transformed to be linear) in many studies although the direction of the trend varied across studies. In the study data, we graphically reviewed the outcome trend in non-transport children, and confirmed that there were no evidences against the analysis plan. (Supplemental figure 3)

3.3 Constant composition

Comparisons by pre- and post-intervention era in each group was listed in the table 1 of the main manuscript. Although there was an increasing trend in home-ventilation dependent and previous PICU

admission over year, most of patient-associated variables were comparable between pre- and post-intervention era.

3.4 Timely enforcement of the intervention

The timely increase in the HHFNC use on interhospital transport following the implementation was confirmed. (Supplemental figure 5)

3.5 No significant effects by temporal variables

Temporal variables were assessed in the final model. Any of temporal variables did not influence the outcome significantly. The estimated outcome effect by the HHFNC implementation in PICU, emergency department, and ward were a ratio of 1.18 (95% CI; 0.95–1.47, p=.12), 1.12 (95% CI; 0.93–1.35, p=.22), and 1.00 (95% CI; 0.84–1.19, p=1.00) based on the final multivariable regression model.

4 Confounders

Confounders to be included in the model were selected from study covariates in the preset method. Multivariable linear regression models with different sets of cofounders were summarized in the supplemental table 4. Across models with various sets of covariates, estimated outcome effects were consistent. Uninfluential variables were chosen from clinical and statistical viewpoints based on specialists' discussion and model fitting by the backward stepwise selection and Akaike information criteria.

5 Other considerations

Seasonality was evaluated by expanding the final mode with indicator variables for each month. The estimated outcome effect by the intervention did not vary much (ratio 0.65, 95% confidence interval (CI) 0.50– 0.84, p=.001).

We performed post-hoc analyses by excluding 802 children with one or more of three underlying conditions (chronic encephalopathy, home-ventilation dependent, and previous PICU admission). The estimated outcome effect in the primary test did not vary much (ratio:0.59, 95% CI:0.44–0.79). When children with each of three underlying conditions were excluded from the analysis, the estimated outcome effect was the ratio (95% CI) of 0.59 (0.45–0.78), 0.65(0.50–0.85), and 0.63 (0.48–0.85), respectively.

Supplemental Table 1 Full list of variables and definitions

Patient-level variables	Definitions
age	age which was categorized in <1, 1–<2, 2–<5, 5–<18 years
Male	1=male, 0=female
Cause of respiratory category	primary respiratory distress on the database categorized in asthma, bronchiolitis, croup, pneumonia, and others
Haemato-oncological disease	1= one or more of underlying haematologic diseases, oncologic disease including leukaemia, lymphoma, haematological disorder, immunodeficiency, previous bone marrow transplant, solid neoplasm, malignant, solid neoplasma
Neuromuscular disease	1= underlying neuromuscular disease including muscular dystrophy, neuropathy, myopathy, myasthenia gravis
Airway disease	1= underlying airway disease including tracheal or bronchial stenosis, tracheal or bronchial malacia, laryngomalacia, choanal atresia or stenosis
Lung disease	1= underlying lung disease including chronic lung disease, cystic fibrosis, bronchiectasis
Chromosomal abnormality	1= chromosomal anomaly including trisomy, monosomy
Chronic encephalopathy	1= underlying encephalopathy chronic static including cerebral palsy, chronic static encephalopathy, chronic degenerative encephalopathy, Leigh's syndrome
Cyanotic congenital cardiac disease	1= presence of cyanotic congenital including hypoplastic left heart syndrome, hypoplastic right ventricle, tricuspid atresia, pulmonary atresia, systemic to pulmonary artery shunt, atrioventricular septal defect, double outlet right ventricle, single ventricle, Ebsteins anomaly etc.
Prematurity	1= gestational week less than 37 weeks among children aged one year or younger on admission
Home-ventilation dependent	1= underlying home-ventilation dependent
Previous PICU admission	1= presence of previous PICU admission within the study period

Month and year	Event
July 2011	Implementation of humidified high-flow nasal cannula in an intensive care unit
January 2012	Increase in the intensive care bed number
April 2013	Implementation of humidified high-flow nasal cannula in an emergency department
January 2014	Implementation of humidified high-flow nasal cannula in paediatric wards
January 2010 to December 2014	Pre-intervention era
January 2015 to December 2019	Post-intervention era

Supplemental Table 2 Comparison of patient characteristics by the source of admission

	Transported, n(%) Transport n=1006		Non-transport, n(%) Non-Transport n=2016		Standardized difference
Age					
<1 year	355	(35.3)	776	(38.5)	0.07
1–<2 years	261	(25.9)	385	(19.0)	0.16
2–<5 years	194	(19.3)	361	(17.9)	0.04
5–<18 years	196	(19.5)	494	(24.5)	0.12
Male, n (%)	616	(61.2)	1206	(59.8)	0.03
Respiratory Category					0.00
Asthma	174	(17.3)	217	(10.8)	0.19
Bronchiolitis	322	(32.0)	715	(35.5)	0.07
Croup	158	(15.7)	149	(7.4)	0.26
Pneumonia	291	(28.9)	891	(44.2)	0.32
Others	61	(6.1)	44	(2.2)	0.20
Haemato-oncological disease	14	(1.4)	58	(2.9)	0.10
Neuromuscular disease	8	(0.8)	49	(2.4)	0.13
Airway disease	33	(3.3)	119	(5.9)	0.13
Lung disease	59	(5.9)	169	(8.4)	0.10
Chromosomal abnormality	43	(4.3)	119	(5.9)	0.07
Chronic encephalopathy	47	(4.7)	342	(17.0)	0.40
Cyanotic congenital cardiac disease	18	(1.8)	48	(2.4)	0.04
Prematurity	170	(16.9)	367	(18.2)	0.03
Home-ventilation dependent	7	(0.7)	100	(5.0)	0.26
Previous PICU admission	93	(9.2)	520	(25.8)	0.45
PIM-2, *median (IQR)*	0.6 (0.2–1.1)		0.7 (0.2–1.2)		0.01

PICU, paediatric intensive care unit; PIM: Paediatric Index of Mortality.

Supplemental Figure 1 Distribution of observed outcomes (n=3022)

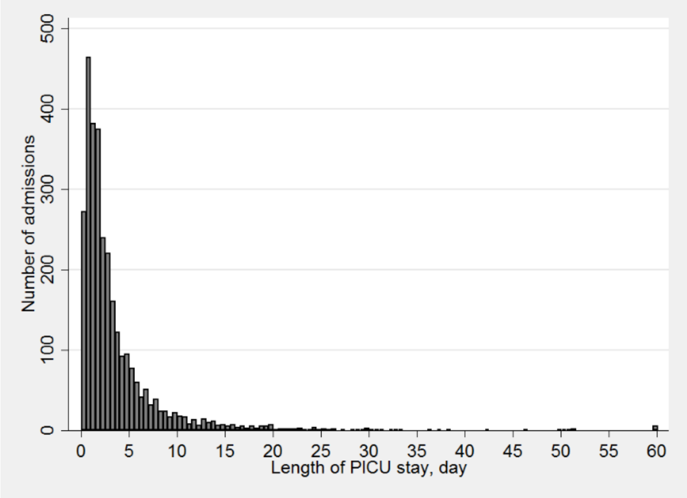

Supplemental Figure 2 Distribution of log-transformed observed outcomes (n=3022)

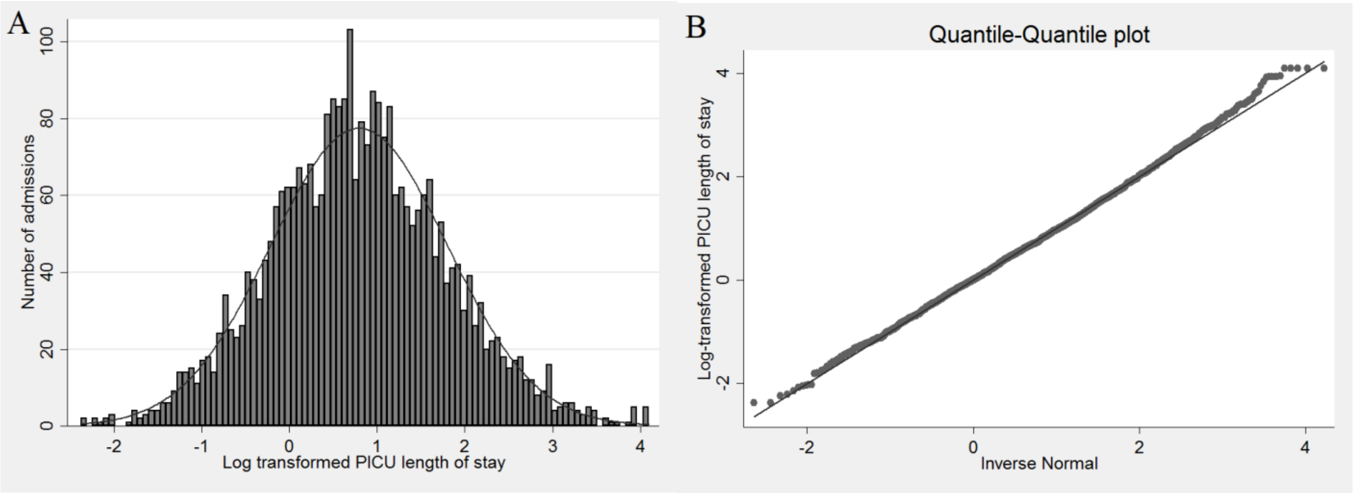

Supplemental Table 3 Regression model fitting

Regression model	Akaike information criteria
Linear regression with a log-transformed outcome	7848.4
Poisson regression	17651.3
Negative binomial regression	13774.6

Supplemental Figure 3 Trend of the observed outcome over year by the source of admission

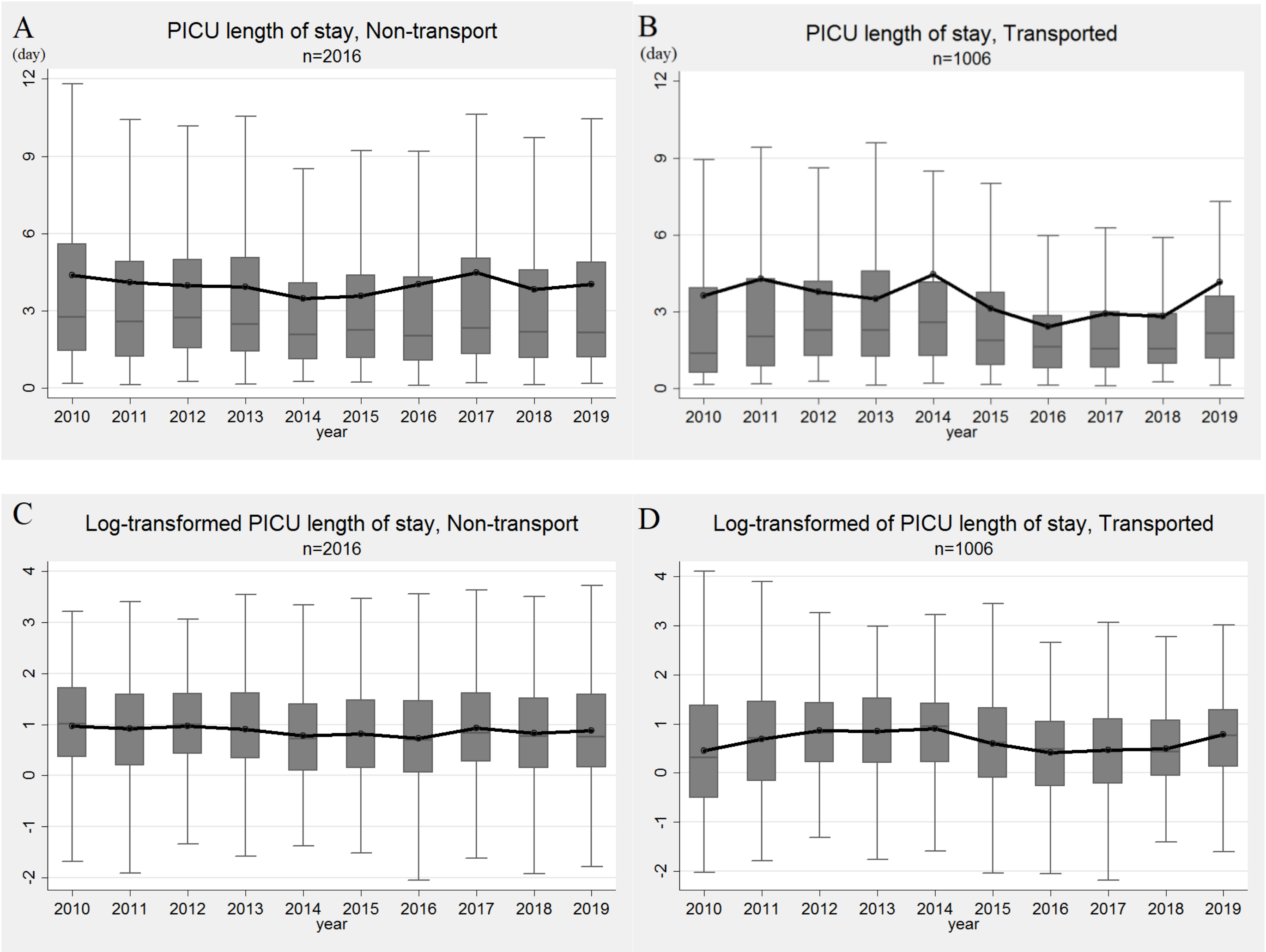

Box plots represent the median, interquartile range, maximal observation below upper fence, and minimal observation above lower fence for each year while connected lines indicate the mean valve.

Supplemental Figure 4 Percentage of humidified high-flow nasal cannula use during interhospital transport (n=1006)

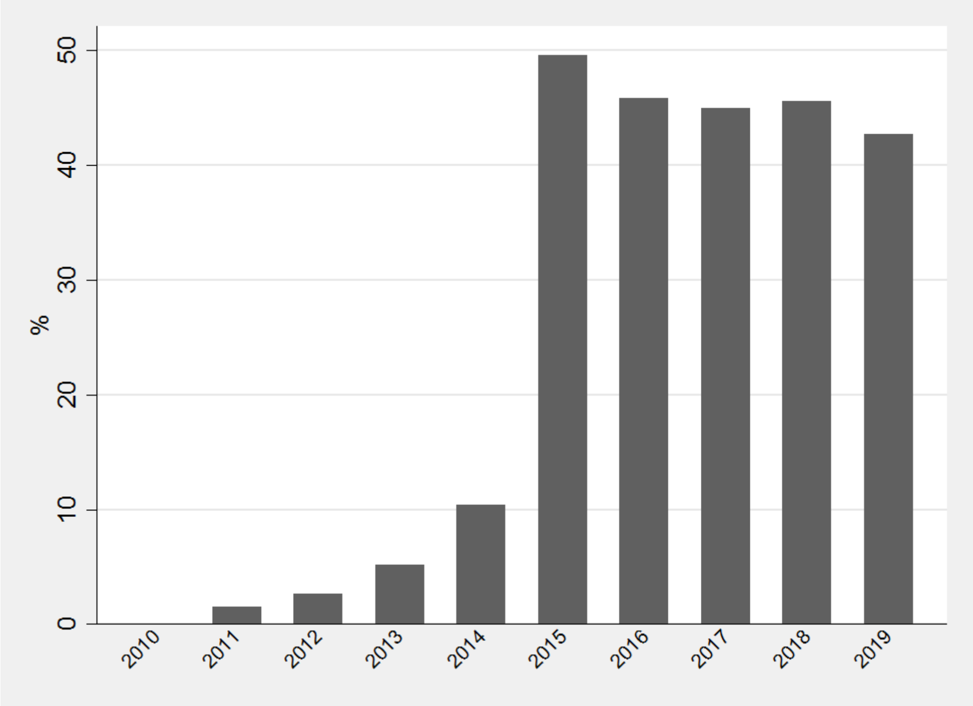

Supplemental Table 4 Models with several set of covariates

Model selection		1<br>Full<br>model	2<br>Final<br>model	3<br>No patient<br>variables	4<br>No timing<br>variables	5<br>No<br>variables
Study design variables[a]		○	○	○	○	○
Timing variables[b]		○	○	○	–	–
Patient-level variables[c]		○	○	–	○	–
Uninfluential variables[d]		○	–	–	–	–
Variables, n		29	26	10	23	7
AIC		7848.4	7845.1	8564.6	7842.8	8568.8
Estimated outcome effect	Ratio (95% CI)	0.64 (0.49–0.83)	0.64 (0.49–0.84)	0.55 (0.41–0.74)	0.65 (0.50–0.84)	0.55 (0.41–0.74)
	p	0.001	0.001	<.001	0.001	<.001

AIC, Akaike Information Criteria; CI, confidence interval.

[a]Study design variables included admission year, transport, post-intervention era, an interaction between transport and post-intervention era, an interaction between year and transport, an interaction between year and post-intervention era, and an interaction between year, post-intervention era and transport.

[b]Timing variables includes three temporal variables for the implementation of high-flow nasal cannula in intensive care unit, emergency department and ward.

[c]Patient-level variables included age category, cause of respiratory distress, sex, haemato-oncological disease, neuromuscular disease, airway disease, lung disease, chromosomal abnormality, chronic encephalopathy, prematurity, home-ventilation dependent, previous PICU admission.

[d]Uninfluential variables included a temporal variable for the incase in intensive care unit bed number, cyanotic congenital cardiac disease, and other types of respiratory diseases.

Supplemental Figure 5 Regression diagnostics

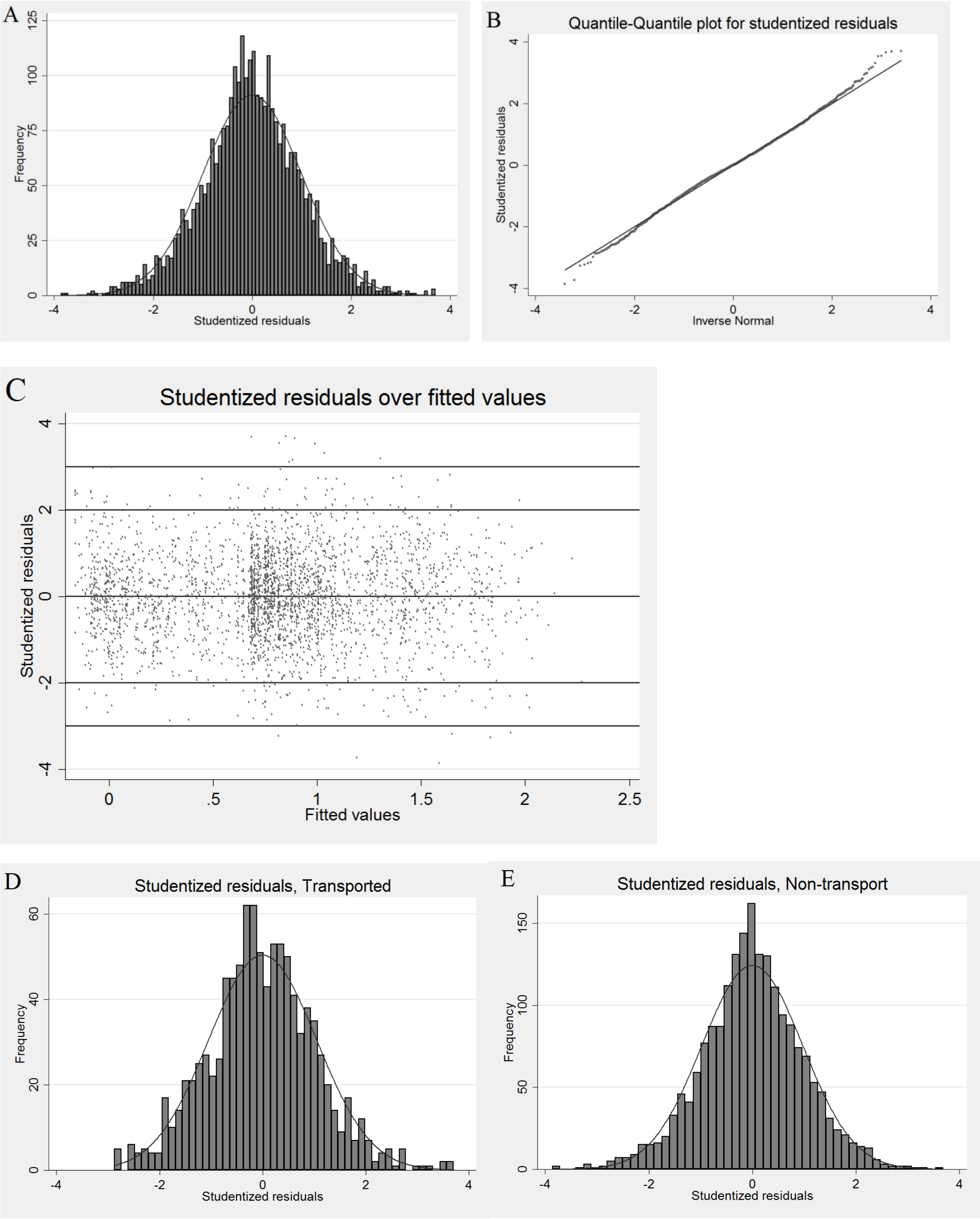

Supplemental Figure 6 Observed outcomes and fitted values based on the final model by admission source

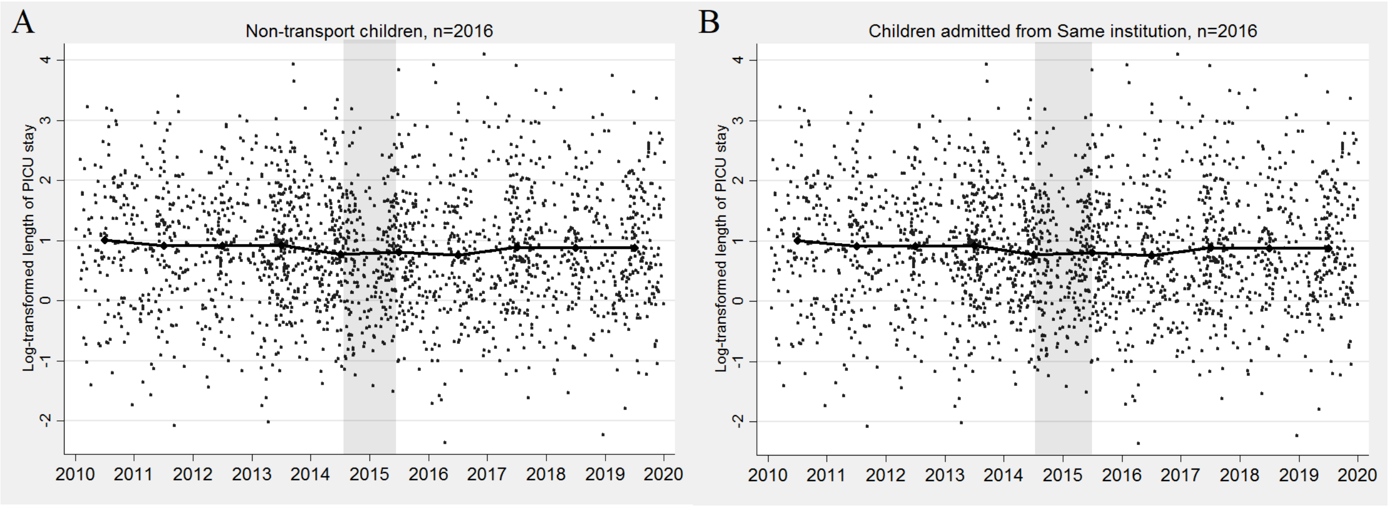

Supplemental Table 5 Trend changes of the length of intensive care unit stay

Cohort	Year	Trend	Trend change between the pre- and post-era	Difference in trend changes between two cohorts	*p*
Transported	2010–2014	0.99 (0.87–1.11)	–	–	
	2015–2019	1.02 (0.97–1.08)	1.03 (0.91–1.19)	0.98 (0.86–1.12)	0.81
Non-transport	2010–2015	0.91 (0.81–1.02)	–	–	
	2015–2020	1.00 (0.97–1.04)	1.1 (0.98–1.25)	–	

–, not applicable

The ratio per year (with 95% confidence interval) was presented.

Supplemental Table 6 Sensitivity analysis with varying outcome effect for each year post intervention

Year	Estimated effect		
	ratio	(95% CI)	*p*
2015	0.74	(0.56–0.99)	0.04
2016	0.55	(0.39–0.78)	0.001
2017	0.49	(0.32–0.73)	<.001
2018	0.54	(0.34–0.87)	0.01
2019	0.56	(0.33–0.96)	0.04

A model extended with interactions between transport and each year post intervention (2015 to 2019) was used to assess whether the effect of the intervention came into the place timely.

Supplementary table 7 Characteristics of propensity-score matched cohorts

	Transported children (n=989), n (%)		Non-transport children (n=989), n (%)		Standardized difference
Age					
<1 year	354	(35.8)	360	(36.4)	0.01
1–<2 years	261	(26.4)	258	(26.1)	<0.01
2–<5 years	186	(18.8)	183	(18.5)	<0.01
5–<18 years	188	(19.0)	188	(19.0)	<0.001
Male	602	(60.9)	608	(61.5)	0.01
Respiratory Category					
Asthma	166	(16.8)	173	(17.5)	0.02
Bronchiolitis	322	(32.6)	337	(34.1)	0.03
Croup	154	(15.6)	140	(14.2)	0.04
Pneumonia	291	(29.4)	302	(30.5)	0.02
Haemato-oncological disease	14	(1.4)	11	(1.1)	0.03
Neuromuscular disease	8	(0.8)	8	(0.8)	<0.001
Airway disease	33	(3.3)	33	(3.3)	<0.001
Lung disease	57	(5.8)	55	(5.6)	<0.01
Chromosomal abnormality	43	(4.3)	38	(3.8)	0.03
Chronic encephalopathy	47	(4.8)	39	(3.9)	0.04
Prematurity	168	(17.0)	171	(17.3)	<0.01
Home-ventilation dependent	7	(0.7)	8	(0.8)	0.01
Previous PICU admission	93	(9.4)	98	(9.9)	0.02
Admission year					
2010	55	(5.6)	50	(5.1)	0.02
2011	66	(6.7)	68	(6.9)	<0.01
2012	77	(7.8)	80	(8.1)	0.01
2013	97	(9.8)	106	(10.7)	0.03
2014	125	(12.6)	132	(13.3)	0.02
2015	123	(12.4)	122	(12.3)	<0.01
2016	126	(12.7)	130	(13.1)	0.01
2017	108	(10.9)	111	(11.2)	0.01
2018	116	(11.7)	111	(11.2)	0.02
2019	96	(9.7)	79	(8.0)	0.06

Supplemental Table 8 Pediatric population in Victoria state

Year	2010	2011	2012	2013	2014
Population, n	1,004,995	1,014,736	1,035,904	1,060,155	1,083,457

Year	2015	2016	2017	2018	2019
Population, n	1,107,752	1,140,064	1,165,231	1,186,110	1,202,099

Supplemental Figure 7 Percentage of transported children with respiratory distress by destination

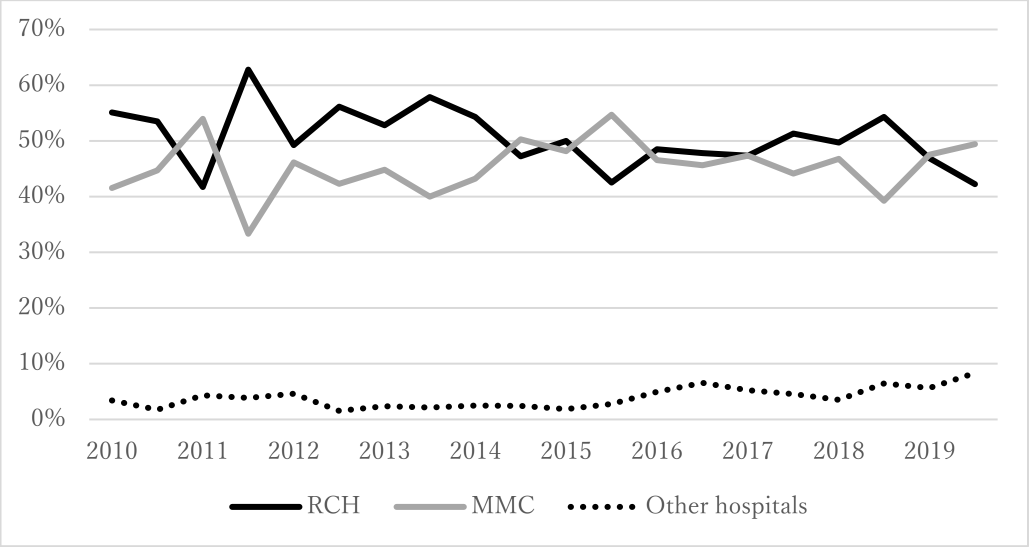

RCH, The Royal Children's Hospital, Melbourne; MMC, Monash Medical Centre, Clyaton.

Supplemental Table 9 The intubation after PICU admission following interhospital transport

Time from admission	2010–2014<br>n=420	2015–2019<br>n=586
Within 4 hours	13 (3.1%)	11 (1.9%)
Within 24 hours	27 (6.4%)	28 (4.8%)
Anytime during PICU stay	35 (8.3%)	37 (6.3%)
