# Supplementary material for: Clinical impact of implementing humidified high-flow nasal cannula on interhospital transport among children admitted to a PICU with respiratory distress: a cohort study
Source: Crit Care. 2021 Jun 6;25:194. doi: 10.1186/s13054-021-03620-7 (PMC8180008; doi:10.1186/s13054-021-03620-7)
Supplement: Supplementary file 3 — Additional file 3. HHFNC protocol on transport. [file 13054_2021_3620_MOESM3_ESM.pdf]

High flow nasal prong (HFNP) oxygen therapy during transport

1. Overview / Description

Humidified high flow nasal prong (HFNP) oxygen therapy is a method for providing oxygen and continuous positive airway pressure (CPAP) to children with respiratory distress. It is used for the same indications as the traditional method of CPAP using a nasopharyngeal tube. HFNP may reduce need for NCPAP/intubation, or provide support post extubation. At high flow of 2 litres per kg per min, using appropriate nasal prongs, a positive distending pressure of 4-8 cmH2O may be achieved. This improves functional residual capacity thereby reducing work of breathing.

2. Related Documents

- PICU High Flow Nasal Prong (HFNP) Therapy
- RCH High Flow Nasal Prong (HFNP) Therapy

3. Definition of Terms

**BiPAP:**	Biphasic Positive Airway Pressure
**CPAP:**	Continuous Positive Airway Pressure
**HFNP oxygen therapy**:	Provision of heated, humidified, blended gas at a flow rate of 2 L/kg/min up to 10kg, plus 0.5 L/kg/min for each kilogram thereafter (to a maximum flow of 50 L/min.)
**WOB**:	Work of breathing
**NIV:**	Non-Invasive ventilation

Responsibility

PIPER Paediatric Medical and Nursing staff.

4. Procedure

4.1 Indications

HFNP oxygen therapy is used for the same indications as the traditional method of CPAP:

- Respiratory distress from bronchiolitis, pneumonia, congestive heart failure, etc.
- Respiratory support post extubation and mechanical ventilation
- Weaning therapy from mask CPAP or BiPAP
- Respiratory support to children with neuromuscular disease
- Apnoea of prematurity

High flow can be used if there is hypoxaemia (SpO_2<90%) and signs of moderate to severe respiratory distress despite standard flow oxygen.

4.2 Contraindications

- Blocked nasal passages/choanal atresia
- Trauma/surgery to nasopharynx

4.3 Management

4.3.1 HFNP Equipment

- Hamilton Ventilator (Figure 1)

  - Select "High Flow" mode
- Humidifier (Fisher and Paykel® MR850) with blue temperature probe and yellow heater cable (Figure 2)

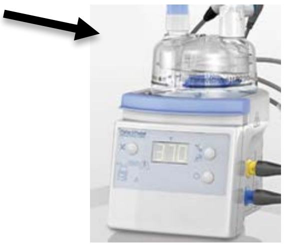

Figure 2

- Circuit tubing (Figure 3)
  - RT380
  - Using Blue Tubing ONLY
  - Keep the rest of the circuit in case of escalation of Respiratory support, e.g. NIV and Invasive Ventilation

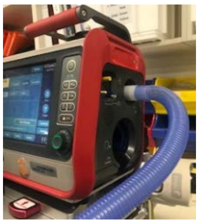

Figure 1

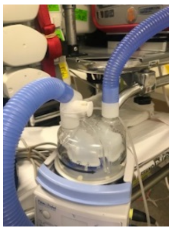

Figure 3

- Nasal prongs (cannula) (size to fit nares comfortably-but not fill the nares)
  - Newborn: OPT312 Premature (red-maximum flow 8L/min) or OPT314 Neonatal (yellow-maximum flow 8L/min)
  - Infants and children up to 10kg: OPT316 Infant (purple- max flow 20L/min)
  - Children >10kg: OPT318 Paediatric Cannula (Max flow 25L/min)
  - Use Green connector 15M-22M to connect RT380 and OPT nasal cannula by removing the white connector from Nasal Cannula
  - Keep the white connector to use it again at a receiving hospital (Figure 4)

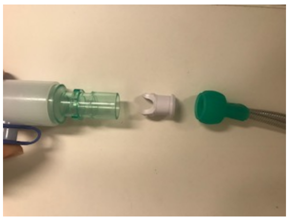

Figure 4

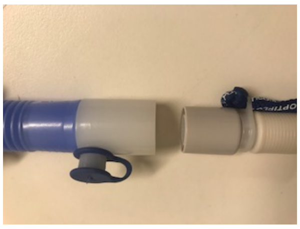

Figure 5

  - Adult cannula size S OPT542, size M OPT544 or size L OPT546 (Figure 5)
- **HFNP nasal prongs are compatible with AIRVO circuits, RT 329 or RT203 circuits.**

4.3.2 <u>Set up of Equipment</u>

- **If HFNP is already initiated at referring hospital,** connect to RT380 circuit. Retain circuit and connector from referring hospital for use at receiving hospital. **If not**, select appropriate size nasal cannula AND circuit for patient size to commence HFNP therapy

  - Place a minimum 50ml sterile water in humidifier chamber and turn on to 37ºC. Do not overfill chamber as it is at increased risk of water entering circuit during transit. Do not use a bag of water if flying as the bag may overfill with air at altitude
- Source sterile water at a referring hospital
- The Fisher and Paykel MR 850 Humidifier is approved for use by Air Ambulance Victoria**.**

4.3.3 Patient Management

- Secure nasal prongs on patient using supplied "wiggle pads™", ensuring the prongs sit well into the nares as needed.
  - Prongs should not totally occlude nares
- Start the high flow nasal cannula system at the following settings:
  - Flow rate
    - ≤10Kg 2 L per kg per minute
    - >10Kg 2 L per kg per minute for the first 10kg + 0.5L/kg/min for each kg above that (max flow 50 L/min)
    - e.g. 16kg= 20L (2 x first 10kg) + 3L (0.5 x 6kg) = 23L/min;
    - 40kg = 20L (2 x first 10kg) + 15L (0.5 x 30kg) = 35L/min
    - Start off at a low flow and increase up to goal flow rate over a few minutes to allow patient to adjust to high flow
  - FiO_2 Start $Fi0_2$ at 0.4-0.5
    - Target range for SpO_2 90%-98%
    - SpO_2 75-85% in cyanotic congenital heart disease with balanced circulation
  - All infants on high flow should have a nasogastric tube (NGT)
  - Humidification
    - Because flows used are high, heated water humidification is necessary to avoid drying of respiratory secretions and for maintaining nasal ciliary function
    - Set humidifier on 37° C invasive setting (length from temperature probe to nares will result in temperature drop to comfortable level whilst maintaining optimal humidity
    - Check humidifier water level hourly
    - Ensure patient's head is higher than the humidifier at all times

4.3.4 Patient Monitoring

- Monitor patient for response
  - Respiratory rate
  - Heart rate
  - WOB
  - SpO_2
- Wean the FiO_2 as tolerated

NOTE:

- Within 2 hours clinical stabilisation should be seen :
  - The FiO_2 required to maintain SpO_2 in the target range (as above) should decrease to <0.4
  - The heart rate and respiratory rate should reduce by 20%
  - Chest in drawing and other signs of respiratory distress should improve
- Hypercarbic respiratory failure may still develop even when oxygen saturations are maintained.
- If there is rapid deterioration of oxygen saturation or marked increased work of breathing, a chest x-ray should be done to exclude a pneumothorax
- Consider different respiratory support if any of the following occurs:
  - The patient is not stabilising as described above
  - The degree of respiratory distress worsens
  - Hypoxaemia persists despite high gas flow
  - Requirement for >0.5 oxygen

4.3.5 Patient Care during transport

- Once stable on high flow, the infant should be assessed as to whether they can feed. Some infants can continue to breast feed, but most require feeding via a nasogastric tube
- Regularly aspirate the NGT 2-4 hourly to avoid abdominal distension
- Ensure nasal prongs are in correct position and no pressure areas to nares
- Gentle suction as required to keep nares clear
- Consider Chloral Hydrate 10-20mg/kg for sedation

5. References

- Dysart K, Miller TL, Wolfson MR, Shaffer TH. (2009) Research in high flow therapy: mechanisms of action. Respiratory Medicine; 103:1400-5.
- Franklin, D., Babl, F., Schlapbach, L., Oakley, E., Craig, S., Neutze, J., Furyk, J., Fraser, J., Jones, M., Whitty, J., Dalziel, S., and Schibler, A. (2018). A Randomized Trial of High-Flow Oxygen Therapy in Infanst with Bronchiolitis. The New England Journal of Medicine 378:1121-31.
- Groves N & Tobin A. (2007). High flow nasal oxygen generates positive airway pressure in adult volunteers. Australian Critical Care. 20, 126-131.
- Kepreotes, E., Whitehead, B., Attia, J., Oldmeadow, C., Collison, A., Searles, A., Goddard, B., Hilton, J., Lee, M. and Mattes, J. (2017). High-Flow Warm Humidified Oxygen vs Standard Low-Flow Nasal Cannula Oxygen for Moderate Bronchiolitis (HFWHO-RCT): an open, phase 4, randomised controlled trial. Lancet389:930-9.
- Spentzas T, Minarik M, Patters AB, Vinson B, Stidham G. (2009) Children with respiratory distress treated with high-flow nasal cannula. J Intensive Care Med; 24:323-8.
- Schibler, A., Pham, T.,Dunster, K., Foster, K., Barlow, A., Gibbons, K., and Hough, J. (2011) Reduced intubation rates for infants after introduction of high-flow nasal prong oxygen delivery. Intensive Care Medicine. May;37(5):847-52.
- McKieman, C., Chua, L.C., Visintainer, P. and Allen, P. (2010) High Flow Nasal Cannulae Therapy in

Infants with Bronchiolitis. Journal of Pediatrics 156:634-38.

6. Disclaimer

The Paediatric, Infant Perinatal Emergency Retrieval (PIPER) Neonatal and Paediatric guidelines were developed by PIPER clinicians for the sole use within the PIPER service at The Royal Children's Hospital Melbourne.

The authors of these guidelines have made considerable effort to ensure the information upon which they are based is accurate and up to date. Users of these guidelines are strongly recommended to confirm that the information contained within them especially drug doses is correct by way of independent resources. The authors accept no responsibility for any inaccuracies or information perceived as misleading.

7. End of Document
